# Methylation of DNA and chromatin as a mechanism of oncogenesis and therapeutic target in neuroblastoma

**DOI:** 10.18632/oncotarget.25084

**Published:** 2018-04-24

**Authors:** Ram Mohan Ram Kumar, Nina Felice Schor

**Affiliations:** ^1^ Department of Pediatrics and Wilmot Cancer Center, University of Rochester School of Medicine and Dentistry, Rochester, NY, USA; ^2^ Current affiliation: National Institute of Neurological Disorders & Stroke, National Institutes of Health, Bethesda, MD, USA

**Keywords:** neuroblastoma, pathogenesis, epigenetics

## Abstract

Neuroblastoma (NB), a developmental cancer, is often fatal, emphasizing the need to understand its pathogenesis and identify new therapeutic targets. The heterogeneous pathological and clinical phenotype of NB underscores the cryptic biological and genetic features of this tumor that result in outcomes ranging from rapid progression to spontaneous regression. Despite recent genome-wide mutation analyses, most primary NBs do not harbor driver mutations, implicating epigenetically-mediated gene regulatory mechanisms in the initiation and maintenance of NB. Aberrant epigenomic mechanisms, as demonstrated by global changes in DNA methylation signatures, acetylation, re-distribution of histone marks, and change in the chromatin architecture, are hypothesized to play a role in NB oncogenesis. This paper reviews the evidence for, putative mechanisms underlying, and prospects for therapeutic targeting of NB oncogenesis related to DNA methylation.

## INTRODUCTION

Neuroblastoma (NB) is among the most common and most deadly solid tumors of childhood. It arises from the neural crest and, although half of neuroblastoma patients have low- or intermediate-risk disease and fare quite well, the remaining 50%, have high-risk NB and only a 40% 5-year survival rate. Only a small minority of NB patients harbor genetic aberrations known to predispose to the disease. There is accumulating evidence that epigenetic factors and mechanisms are responsible for the malignancy and therapeutic resistance of NB in most patients [1, 2].

The introduction of targeted therapies to chemotherapy regimens has made sufficient impact on overall survival in patients with NB. It has become evident that the cancer epigenomic landscape contributes significantly to the pathogenesis of NB and may play an important role in initiation of tumorigenesis. Epigenetic therapies such as DNA methyltransferase inhibitors and histone deacetylase inhibitors have the potential to reverse these epigenetic changes [3]. However, further research on NB epigenetics and the targeting of the activity of epigenetic enzymes at specific genes and sequences is needed to determine how to incorporate these agents into clinical practice. In this review, we focus on DNA methylation events, currently the most widely studied and best understood stable epigenetic modifications, and epigenetic therapy in NB. We also specifically discuss the role of chromatin methylation in regulation of MYCN, a prognostically important oncogene in NB.

## DNA METHYLATION IN NB

DNA methylation, an essential epigenetic mechanism, is closely correlated with the processes underlying cell growth, differentiation and transformation [3]. DNA methylation attenuates gene expression by the addition of methyl groups to cytosine residues within CpG-rich sequences present in the promoter region of genes. Even though details of the regulatory mechanisms that result from DNA methylation remain to be elucidated, the regulatory effects of methylation on gene expression patterns in many cancers have been identified. For instance, aberrant DNA methylation at promoter CpG islands is generally associated with reduced transcriptional activity and silencing of tumor suppressor genes, thereby contributing to induction of malignancy [4–6]. However, DNA methylation at non-CpG sites has also been reported in cancer cells and studies by Gómez *et al.* in a small cohort of patients with NB identified the presence of non-CpG methylation sites and their association with differentiation and expression of some of the key genes involved in NB, including ALK [7]. Nonetheless, during oncogenesis, methyl groups at non-CpG sites are asymmetrical and not widely distributed, and methylated CpG sites still represent the main epigenetic determinants in cancer. Genome-wide DNA methylation analyses are now increasingly employed to determine the epigenetic events involved in NB tumorigenesis.

### DNA methylation profiling of primary NB tumors and cell lines

To identify epigenetic deregulation mechanisms in NB tumorigenesis, Charlet *et al.* compared the methylation pattern of NB cell lines to that of human neural crest precursor cells, using promoter and CpG island microarrays. In their analysis, among hypermethylated genes, MEGF10, a cell engulfment and adhesion factor gene, was epigenetically repressed in the NB cell lines. MEGF10 expression was also found to be significantly downregulated in NB tumor samples, a circumstance associated with reduced relapse-free survival. Also, knockdown of MEGF10 in NB cell lines *in vitro* promoted cell growth and proliferation, suggesting it harbored an epigenetic mark that maintained the silenced state of this gene [8]. *In vivo* studies are needed to validate the effect of MEGF10 in NB. Methylation patterns can also be used to divide NB tumors into clinically relevant groups. In a study by Olsson *et al.*, methylation at the CpG sites of a large number of genes was found in metastatic NB. Telomerase reverse transcriptase (TERT) had the highest number of hypermethylated sites and was found to be associated with tumor progression and poor prognosis in NB. Methylation of the TERT gene has been proposed as a biomarker for risk stratification of NB [5]. Rauschert and colleagues reported an epigenetic mechanism underlying the silencing of Lamin A/C, which supports nuclear assembly, chromatin organization, and telomere dynamics, by CpG promoter hypermethylation in a subset of NB cells, leading to enhanced tumor aggressiveness. The effect of hypermethylation was evident when Lamin A/C was reintroduced in the cell lines deficient in this gene, which induced slowing of cell growth kinetics: delayed migration, invasion, and colony formation, together with cytoskeletal reorganization [9]. Enhancing expression of Lamin A/C in NB might represent a therapeutic strategy, since tumor cells deficient in Lamin A/C exhibit more aggressive behavior. In a recent study by Henrich *et al.*, Protocadherin Beta Cluster (PCDHB) methylation patterns were identified in the tumors of patients who had high-risk NB. Hypermethylation of PCDHB correlated with methylation of intragenic enhancer elements and with hypomethylation and enhanced expression of genes associated with the biology of aggressive neuroblastoma [10].

Among different stages of NB, special stage IV (Stage 4S) NB presents an intriguing condition in which infants diagnosed with metastases have an unusually excellent outcome, due to the tumor’s ability to undergo spontaneous regression [11]. In order to understand the phenomenon of spontaneous regression, Decock *et al.* profiled the promoter methylome of stage 4S NB patients using methyl-CpG-binding domain (MBD) sequencing analysis in primary tumor samples. The DNA methylation pattern of stage 4S NB, when compared with stage 1/2 and stage 4 NB, is dominated by differential methylation of target genes of several transcription factors that are involved in neural crest development and neural differentiation, like MSX1, EVI1, E2F1, EGR3, AHR, MEF2A, YY1, PPARA, POU2F1 and GFI1. SLC9A5, a target gene of E2F1 and MEF2A, was found to be hypermethylated in stage 4S when compared to stage 1/2 and stage 4 tumors [12]. While this study did not define the exact mechanisms by which DNA methylation contributes to differential regulation of oncogenic signalling pathways in stage 4S NB, it does make clear the biological difference between stage 4S and other low-risk NBs.

A feature of high-risk NB is the high level of DNA methylation of putative tumor suppressor genes. Studies on a number of NB candidate genes have revealed that this hypermethylation drives the NB oncogenic process [6]. Epigenetic silencing of tumor suppressor genes, including RASSF gene family members, RASSF2, RASSF4, RASSF5, RASSF6, RASSF7, and RASSF10, has been found to be frequent in NB cell lines and primary tumors and is considered to be involved mediating poor prognosis. Since promoter hypermethylation of RASSF gene family members modulates the growth inhibitory responses mediated by Ras, detection of RASSF can play a role in NB prognosis and treatment. Treatment with 5-Aza-dC (DAC), an epigenetic modifier that inhibits DNA methyltransferase activity, in NB cell lines restored the RASSF gene expression by blocking RAS-induced apoptosis [13]. Several tumor suppressor genes have reported to be epigenetically silenced by DNA methylation in NB tumor and cell lines; these findings are outlined in Table 1.

**Table 1:** Summary of tumor suppressor genes silenced by promoter methylation in NB

Genes	Functional significance	Methylation effects in NB	Methylation patterns (%)		References
			Cell lines	Primary tumors	
*CASP8*	Involved in the programmed cell death induced by Fas	Inactivation of Fas	56-92%	14-91%	[14,15]
*CD44*	Cell surface molecule involved in cell proliferation, cell differentiation, cell migration, angiogenesis	Disruption of cell-cell interaction, cell adhesion and migration	33%	82%	[16]
*CADM1*	Involved in cell-cell adhesion in a Ca (2+)-independent manner	Deregulated neuronal migration	-	30.2%	[17]
*PHOX2B*	Active in the neural crest and helps in neuronal differentiation	Deregulated neuronal migration	76.9%	88.2%	[18]
*TMS1*	Involved in apoptosis and inflammasomes function	Inhibits apoptotic signalling pathway	-	25%	[19]
*BLU*	Tumor suppressor gene	Increased proliferation and migration of NB cells	21%	7%	[20, 21]
*EMP3*	Inhibits cell proliferation, cell-cell interactions and function as a tumor suppressor	Increased proliferation and migration	-	24%	[22]
*SFRP1*	Act as soluble modulators of Wnt signalling pathway	Deregulated activation of Wnt pathway	27%	27%	[23]
*PTCH1*	Tumor suppressor gene. Acts as a receptor for sonic hedgehog (SHH)	Deregulated activation of Wnt pathway	27%	22%	[23]
ABCB1	Actively involved in the efflux of antineoplastic agents from cancer cells.	Dysregulated drug resistance	80%	-	[24]

### Biomarkers of NB identified by methylation profiling

Whole-genome methylation profiling of NB tumors with a high degree of clinical heterogeneity, matched with normal tissue from NB patients, has led to the identification of a large number of putative prognostic biomarkers, but only very few have shown clinical validity and utility due to inadequate study design, insufficient cohort size and consequent statistical power, and lack of biomarker validation, thus falling short of being able to predict NB prognosis [25]. Many of these studies performed methylation profiling in efforts to identify prognostic biomarkers in NB and establish the connection between epigenetic events and NB progression. However, the number of comprehensive genome-wide DNA methylation studies that analyze primary tumor samples for biomarker identification in NB is limited. Furthermore, MYCN expression, one of the best-characterized biomarkers in NB, does not fully reflect disease prognosis and has not been identified as an epigenetically-regulated biomarker by methylation profiling [14]. Decock *et al.* performed methyl-CpG-binding domain sequencing analysis in 87 primary tumors, a larger number of independent samples than used in other reported studies, and two independent cohorts of 132 and 177 primary tumors were used to identify NB-specific prognostic biomarkers. They identified novel prognostic methylation biomarkers: CCDC177, NXPH1, SPRED3, TNFAIP2, NPM2 for NB event-free survival and CYYR1 for overall survival. Interestingly, most of the genes identified in the analysis are linked to neurite outgrowth [26]. Table 2 lists the prognostic biomarkers of NB identified by methylation profiling.

**Table 2:** List of prognostic methylated biomarkers identified in NB

Methylated gene (s)	Functional significance	Prognostic predictor	References
*FOLH1*	Functions as protection from apoptosis or degradation of brain neurons	Poorer clinical outcome, independent of MYCN amplification	[27]
*MYOD1*	Regulates muscle cell differentiation by inducing cell cycle arrest	Poorer clinical outcome, independent of MYCN amplification	[27]
*THBS1*	Involved in cell-to-cell and cell-to-matrix interactions	Poorer clinical outcome, independent of MYCN amplification	[27]
*FOXP1*	Acts as tumor suppressor and transcriptional repressor	Unfavourable patient outcome	[28]
*RB1*	Tumour suppressor gene. Prevent excessive cell growth by inhibiting cell cycle progression	Poor survival	[29]
*TDGF-1*	Plays an essential role in embryonic development and tumor growth	Poor survival	[29]

### miRNAome methylation in NB

miRNAs are highly conserved and involved in many biological processes including cell proliferation, apoptosis, migration and differentiation. In a pathologic environment, miRNA dysregulation contributes to phenotypic alterations, mimicking the function of tumor suppressor genes or oncogenes according to the function of the proteins encoded by the target gene [30]. One of the characteristic features of miRNA is that it can influence gene expression without altering the DNA sequence, making it an integral component of the epigenetic machinery. DNA methylation that results in the hypo- or hypermethylation of miRNAs indirectly influences the up- or down-regulation of target genes, respectively [31]. Coordinated actions of miRNAs and other epigenetic factors regulate several biological processes in which miRNAs can repress the expression of epigenetic factors or cooperate to modulate common targets. Most miRNA genes have CpG sites and are regulated by DNA methylation in tumors and in cancer-specific conditions, exemplified by miR-31 in breast cancer [32].

Parodi *et al.* studied the complex network of miRNAs and genes involved in cell cycle and apoptosis pathways in NB. DNA methylation screening in regulatory regions of miRNAs involved in those pathways revealed potential methylation targets in NB, namely, cluster 34b/c, cluster 23b/24-1/27b, miR-124, miR-149, miR-155 and miR-196a1 in NB cell lines. DNA methylation analysis in tumor samples of NB patients also confirmed the presence of hypermethylation for cluster 34b/c and miR-124, which might play a role in NB aggressiveness. This study revealed the presence of epigenetic dysregulation, which contributed to the functionality of cell cycle and activation of the apoptosis pathway in NB [33]. Maugeri *et al.* investigated the role of promoter methylation in miRNAs encoding genes in NB. They profiled 754 miRNAs of specific CpG islands using methylation assays and *in silico* analyses. miR-29a-3p, miR-34b-3p, miR-181c-5p and miR-517a-3p were found to be methylated and the promoter encoding miR-29a-3p, which is known to be down-regulated in NB, has methylated CpG islands which decreased on treatment with 5’-AZA, suggesting that increased expression of miRNA is linked to decreased NB cell viability [34]. Functional studies have determined that several of the hypermethylated miRNAs, listed in Table 3, target a large repertoire of genes that are over-expressed in NB tumors with substantial redundancy and collectively negatively impact NB cell proliferation and migration, both *in vitro* and *in vivo*. Das *et al.* investigated the role of coordinated miRNA and DNA methylation changes in regulating NB cell differentiation by using all trans-retinoic acid (ATRA), which causes NB cell lines to extrude and elongate neurites during the process of neural cell differentiation. They identified demethylation of methyl-transferase genes, DNTMT1 and DNTMT3, along with upregulation of miRNAs targeting them, such as miR-152 and miR-26a/b, following the ATRA treatment [35]. Table 3 lists the hypermethylated miRs and their target genes identified in NB.

**Table 3:** Hypermethylated miRs and their targets in NB

Hypermethylated miRs	Target genes	References
miR-340	*SOX2*	[35]
Let-7, miR-101	*MYC*	[36]
miR-335	*TGFβ, MAPK1, LRG1, ROCK1*	[37, 38]
miR-184	*AKT2*	[39]
miR-137	*CAR*	[40]

## EPIGENETIC THERAPY IN NB

As normal cells undergo malignant transformation, epigenetic modifiers such as DNA methyltransferases (DNMT) and histone deacetylases (HDACs) maintain the modification status of gene loci in tumor cells [41]. Studies using demethylating agents and HDAC inhibitors demonstrate that genes such as tumor suppressor genes can be re-expressed in cell lines, but the impact of these agents in clinical settings is not yet clear. Despite many challenges, on-going clinical trials are aimed at identifying the potential role of epigenetic therapies in NB. At present, this approach is not yet considered standard of care and a combination of such agents with conventional chemotherapeutic drugs might improve sensitivity of NB to chemotherapy.

### Drugs targeting DNA methylation in NB

Unlike genetic alterations, DNA methylation can be reversed to restore the function of key control pathways in malignant and premalignant cells. For example, treatment with demethylating agents such as DNA methyltransferase inhibitors (DNMTi) namely azacitidine (5-azacitidine) and decitabine (5-Aza-deoxycytidine; AZA) induces functional reversion of aberrantly silenced genes in cancer [39]. These classes of inhibitors are now being evaluated in Phase 1 clinical trials in combination with other agents in patients with NB. AZA has been studied in clinical trial as an anticancer drug for patients with NB, but was not well tolerated because of its severe myelosuppressive effects and narrow therapeutic window vis-à-vis activation of tumor suppressor genes [42]. One of the limitations of AZA in NB is it is a poor activator of tumor suppressor genes. Despite this, studies by Westerlund *et al.* combined AZA and the differentiation-promoting drug retinoic acid (RA) and thereby impeded NB growth and induced the expression of HIF2α, a tumor suppressor gene. This combination approach targeted high-risk NB that responded poorly to RA therapy alone [43]. Another group reported on treatment with AZA and tamibarotene (TBT), a synthetic retinoid, in a panel of NB cell lines; this regimen supressed proliferation and induced an increase in the number of cells in S phase. The combination of AZA and TBT was also investigated *in vivo* in a mouse xenograft model; it resulted in significant tumor regression without severe side effects [44].

### Drugs targeting changes in histone methylation state in NB

Well-known histone modifications that are found to be involved in regulating gene expression include methylation, demethylation, acetylation, and deacetylation. Histone methylation (HM) is involved in gene transcription and chromatin remodelling and is linked to inactivation of a number of critical tumor suppressor genes. HM is considered to be an epigenetic mark that is dynamically regulated by histone methyltransferases and demethylases. Histone methyltransferases (HMT), which catalyze histone methylation, are widely studied chromatin-modifying enzymes and are considered to be a potential therapeutic target. Numerous studies have been reported for HMT inhibitors in NB cell lines. These studies demonstrate the effect of these agents on cell proliferation and migration. In one recent study, treatment with the small molecule inhibitor, SGC0946, which targets DOT1L, a histone methyltransferase that catalyzes methylation at the H3K79 position, reduced H3K79 methylation and down-regulated MYCN, ODC1, and E2F2 genes, reducing NB cell proliferation [45]. However, *in vivo* studies are needed to determine the efficacy of these inhibitors of HM in NB. Recently, Veschi *et al.* identified SETD8, a methyl transferase which catalyzes methylation of H4K20, as a crucial regulator of cell growth and differentiation in high-risk NB. Pharmacological inhibition of SETD8 by UNC0379 in NB cell lines induced SETD8 knockdown and effectively inhibited the proliferation of cells *in vitro* and in *ex vivo* models [46]. Ke *et al.* studied the inhibition of G9a, a methyltransferase for H3K9, by BIX01294 in a xenograft mouse NB mouse model. Treatment with BIX01294 resulted in reduced tumor volume in NOD/SCID mice, raising the possibility of therapeutically targeting G9a in NB [47].

Histone demethylases (HDM) induce the expression of oncogenic transcription factors including MYC [48]. HDM family members with diverse functions are implicated in regulation of NB cell survival. Targeting HDM may block the expression of oncogenic transcription factors like MYC and activate tumor-suppressive pathways in NB. Recently, Yang *et al.* identified the novel histone demethylase inhibitor, ciclopirox, that binds KDM4B, an HDM that is one of a family of lysine demethylases, and inhibited NB growth and metastasis in a disseminated disease model of NB [49]. The study indicates that pan-KDM inhibition in NB clinical trials might contribute to its overall anti-tumor effect. Another KDM, LSD1, physically binds to MYCN both *in vitro* and *in vivo*. Combined pharmacological inhibition of MYCN and LSD1 by TCP and 10058-F4, respectively, reduced MYCN-amplified NB cell viability *in vitro*. The ability of these inhibitors to specifically inhibit the function of both MYCN and LSD1 is of great importance and could lead to development of novel therapeutic approaches to treat MYCN-induced NB [50].

HDACs enzymatically remove the acetyl group from histones and regulate gene expression. HDAC inhibitors are a class of epigenetic modifiers that activate silent genes such as cyclin-dependent kinase by altering the acetylation state of their histone tails [41]. HDAC inhibitors block the activity of HDAC isozymes involved in numerous biological processes and the potential for toxicities that result in dose-limiting side effects were reported for pa n-HDAC inhibition [51]. Rettig and colleagues reported selective inhibition of one member of the HDAC family, HDAC8, which is highly expressed in metastasized NB tumors; this approach was shown to be effective and less toxic than the non-specific inhibition of several HDAC family members in a preclinical model of NB [52]. HDAC-selective targeting might be an effective therapeutic strategy in tumors that express HDAC isozymes and could be combined with differentiation-inducing agents like RA. One group investigated the potential activity of an HDAC inhibitor, MS-275, in combination with a pan-carbonic anhydrase inhibitor, acetazolamide (AZ) in a pre-clinical NB xenograft model. On co-treatment, cancer stem cell genes (OCT4, SOX2 and NANOG) were found to be down-regulated, which indicated the elimination of the NB-cancer stem cell properties. The combination treatment drastically reduced tumor growth *in vivo* and suggests the therapeutic potential of HDAC inhibitors in patients with NB [53]. Combination studies of RA with the HDAC inhibitor trichostatin A, (TSA), resulted in anti-tumorigenic effect in SH-SY5Y and SK-N-BE cells, suggesting that this combined therapy could be useful to inhibit NB progression [54].

Histone acetylation is important in differentiation and proliferation, signal transduction, metabolism and cytoskeleton dynamics and initiated by the activity of histone acetyltransferases (HATs), involved in acetylating conserved lysine residues by transferring an acetyl group from acetyl-CoA to form N-acetyl-lysine. Histone lysine acetylation is involved in epigenetic modifications that impact gene expression and transcriptional activity [55]. The pyridoisothiazolone HAT inhibitors, PU139 and PU141, have been found to induce cellular histone hypoacetylation and inhibit growth of NB cell lines. Both of these agents were able to block growth of SK-N-SH NB xenografts in mice due to their reduction of histone lysine acetylation [56]. The effect of these agents needs to be studied in clinical trials in NB patients.

## EPIGENETIC REGULATION OF MYCN EXPRESSION AND N-MYC STABILITY AS TARGETS FOR THERAPY OF NB

The most extensively studied arbiters of prognosis in NB are the oncogene MYCN and its gene product, N-Myc. Amplification of MYCN correlates with poor prognosis and is most often seen in advanced-stage disease. N-Myc is a transcription factor, the downstream targets of which include ALK, gene mutations of which convey aggressive behavior upon NBs. The downstream targets of N-Myc are so numerous and varied in effect on the cell as to make direct targeting of N-Myc, even for a subset of aggressive NBs, therapeutically intractable. For this reason, there has been an experimental focus on discerning the interactions of N-Myc with other proteins that lead specifically to increased malignancy of NB [57].

Chromatin methylation plays direct and indirect roles in the regulation of MYCN expression and the stability of MYCN. For example, among the proteins that regulate MYCN expression is the HDM, KDM4B. The N-Myc protein forms a complex with KDM4B that binds to N-Myc responsive genes, including MYCN. The prevalence of this complex in NB correlates with degree of malignancy [58].

The RNA, lncUSMycN, forms a complex with the RNA-binding protein, NonO, that induces MYCN expression. In addition, lncUSMycN upregulates expression of the MYCN antisense gene, NCYM; conversely, knockdown of lncUSMycN reduces histone H3 lysine 4 trimethylation, a marker for active gene transcription, at the NCYM promoter. NCYM also upregulates MYCN transcription and knocking down NCYM expression, e.g., with BET bromodomain inhibitors, reduces NB cell proliferation. In children with NB, high levels of NCYM expression in tumor tissues correlated with high levels of N-Myc, NonO, and lncUSMycN expression and poor patient prognosis [59]. NCYM has also been shown to stabilize N-Myc protein by inhibiting the N-Myc-degradative activity of the kinase GSK3β. Clinically, NCYM plays an essential role in NB cell metastasis [60].

N-Myc complexes with WD repeat-containing protein 5 (WDR5) and histone H3K4 facilitating trimethylation of H3K4 and resulting in transcriptional activation of genes, including MDM2, involved in tumorigenesis in NB [61]. Similarly, N-Myc recruits HDAC5 to the CD9 gene, repressing CD9 protein expression and decreasing CD9 glycoprotein content of the cell membrane, thereby increasing the degree of malignancy of clinical NB. N-Myc is also a transcriptional repressor of the GHRL1 gene, the gene product of which activates CD9 expression [62].

The study of genes other than MYCN amplification or mutation of which correlates with the degree of malignancy of NB has identified downstream effects in common among oncogenic species and suggested the final common pathway to aggressiveness of clinical NB. TERT rearrangement occurs exclusively in high-risk NB and defines a particularly poor prognosis subgroup of these tumors. Massive chromatin remodelling and DNA methylation are seen in tumors with TERT rearrangement and lead to telomere lengthening, a phenomenon also seen in high-risk NB-associated MYCN amplification and ATRX mutations. This suggests that telomerase activation and consequent telomere lengthening may be at the center of the virulence of NB [63].

## CONCLUSIONS

The integration of genomic and epigenetic data provides strong evidence that methylation of DNA and chromatin is highly dysregulated in NB. These aberrantly regulated epigenetic processes interact with one another, rather than operating independently, thereby establishing a multilevel regulatory network altering the expression of tumor-suppressive, cell cycle-active, and cell survival determinant genes. An increased understanding of the epigenetic phenomena that drive NB will suggest novel avenues for prevention and treatment of this deadly tumor of childhood, and extensive basic and clinical studies are required to translate these findings into favorable patient outcome.

## References

[1] Ratner N, Brodeur GM, Dale RC, Schor NF (2016). The “neuro” of neuroblastoma: neuroblastoma as a neurodevelopmental disorder. Ann Neurol.

[2] Ganeshan VR, Schor NF (2011). Pharmacologic management of high-risk neuroblastoma in children. Paediatr Drugs.

[3] Rodríguez-Paredes M, Esteller M (2011). Cancer epigenetics reaches mainstream oncology. Nat Med.

[4] Jones PA, Baylin SB (2002). The fundamental role of epigenetic events in cancer. Nat Rev Genet.

[5] Olsson M, Beck S, Kogner P, Martinsson T, Carén H (2016). Genome-wide methylation profiling identifies novel methylated genes in neuroblastoma tumors. Epigenetics.

[6] Esteller M (2002). CpG island hypermethylation and tumor suppressor genes: a booming present, a brighter future. Oncogene.

[7] Gómez S, Castellano G, Mayol G, Suñol M, Queiros A, Bibikova M, Nazor KL, Loring JF, Lemos I, Rodríguez E, de Torres C, Mora J, Martín-Subero JI, Lavarino C (2015). DNA methylation fingerprint of neuroblastoma reveals new biological and clinical insights. Epigenomics.

[8] Charlet J, Tomari A, Dallosso AR, Szemes M, Kaselova M, Curry TJ, Almutairi B, Etchevers HC, McConville C, Malik KT, Brown KW (2017). Genome-wide DNA methylation analysis identifies MEGF10 as a novel epigenetically repressed candidate tumor suppressor gene in neuroblastoma. Mol Carcinog.

[9] Rauschert I, Aldunate F, Preussner J, Arocena-Sutz M, Peraza V, Looso M, Benech JC, Agrelo R (2017). Promoter hypermethylation as a mechanism for Lamin A/C silencing in a subset of neuroblastoma cells. PLoS One.

[10] Henrich KO, Bender S, Saadati M, Dreidax D, Gartlgruber M, Shao C, Herrmann C, Wiesenfarth M, Parzonka M, Wehrmann L, Fischer M, Duffy DJ, Bell E (2016). Integrative Genome-Scale Analysis Identifies Epigenetic Mechanisms of Transcriptional Deregulation in Unfavorable Neuroblastomas. Cancer Res.

[11] Taggart DR, London WB, Schmidt ML, DuBois SG, Monclair TF, Nakagawara A, De Bernardi B, Ambros PF, Pearson AD, Cohn SL, Matthay KK (2011). Prognostic value of the stage 4S metastatic pattern and tumor biology in patients with metastatic neuroblastoma diagnosed between birth and 18 months of age. J Clin Oncol.

[12] Decock A, Ongenaert M, De Wilde B, Brichard B, Noguera R, Speleman F, Vandesompele J (2016). Stage 4S neuroblastoma tumors show a characteristic DNA methylation portrait. Epigenetics.

[13] Djos A, Martinsson T, Kogner P, Carén H (2012). The RASSF gene family members RASSF5, RASSF6 and RASSF7 show frequent DNA methylation in neuroblastoma. Mol Cancer.

[14] Teitz T, Wei T, Valentine MB, Vanin EF, Grenet J, Valentine VA, Behm FG, Look AT, Lahti JM, Kidd VJ (2000). Caspase 8 is deleted or silenced preferentially in childhood neuroblastomas with amplification of MYCN. Nat Med.

[15] Casciano I, Banelli B, Croce M, De Ambrosis A, di Vinci A, Gelvi I, Pagnan G, Brignole C, Allemanni G, Ferrini S, Ponzoni M, Romani M (2004). Caspase-8 gene expression in neuroblastoma. Ann N Y Acad Sci.

[16] Yan P, Mühlethaler A, Bourloud KB, Beck MN, Gross N (2003). Hypermethylation-mediated regulation of CD44 gene expression in human neuroblastoma. Genes Chromosomes Cancer.

[17] Nowacki S, Skowron M, Oberthuer A, Fagin A, Voth H, Brors B, Westermann F, Eggert A, Hero B, Berthold F, Fischer M (2008). Expression of the tumour suppressor gene CADM1 is associated with favourable outcome and inhibits cell survival in neuroblastoma. Oncogene.

[18] de Pontual L, Trochet D, Bourdeaut F, Thomas S, Etchevers H, Chompret A, Minard V, Valteau D, Brugieres L, Munnich A, Delattre O, Lyonnet S, Janoueix-Lerosey I, Amiel J (2007). Methylation-associated PHOX2B gene silencing is a rare event in human neuroblastoma. Eur J Cancer.

[19] Grau E, Martinez F, Orellana C, Canete A, Yañez Y, Oltra S, Noguera R, Hernandez M, Bermúdez JD, Castel V (2011). Hypermethylation of apoptotic genes as independent prognostic factor in neuroblastoma disease. Mol Carcinog.

[20] Kiss NB, Kogner P, Johnsen JI, Martinsson T, Larsson C, Geli J (2012). Quantitative global and gene-specific promoter methylation in relation to biological properties of neuroblastomas. BMC Med Genet.

[21] Agathanggelou A, Dallol A, Zöchbauer-Müller S, Morrissey C, Honorio S, Hesson L, Martinsson T, Fong KM, Kuo MJ, Yuen PW, Maher ER, Minna JD, Latif F (2003). Epigenetic inactivation of the candidate 3p21.3 suppressor gene BLU in human cancers. Oncogene.

[22] Fumoto S, Tanimoto K, Hiyama E, Noguchi T, Nishiyama M, Hiyama K (2009). EMP3 as a candidate tumor suppressor gene for solid tumors. Expert Opin Ther Targets.

[23] Shahi MH, Schiapparelli P, Afzal M, Sinha S, Rey JA, Castresana JS (2011). Expression and epigenetic modulation of sonic hedgehog-GLI1 pathway genes in neuroblastoma cell lines and tumors. Tumour Biol.

[24] Qiu YY, Mirkin BL, Dwivedi RS (2007). MDR1 hypermethylation contributes to the progression of neuroblastoma. Mol Cell Biochem.

[25] Cohn SL, Pearson AD, London WB, Monclair T, Ambros PF, Brodeur GM, Faldum A, Hero B, Iehara T, Machin D, Mosseri V, Simon T, Garaventa A, INRG Task Force (2009). The International Neuroblastoma Risk Group (INRG) classification system: an INRG Task Force report. J Clin Oncol.

[26] Decock A, Ongenaert M, Cannoodt R, Verniers K, De Wilde B, Laureys G, Van Roy N, Berbegall AP, Bienertova-Vasku J, Bown N, Clément N, Combaret V, Haber M, Children’s Cancer and Leukaemia Group (CCLG) (2016). Methyl-CpG-binding domain sequencing reveals a prognostic methylation signature in neuroblastoma. Oncotarget.

[27] Lau DT, Hesson LB, Norris MD, Marshall GM, Haber M, Ashton LJ (2012). Prognostic significance of promoter DNA methylation in patients with childhood neuroblastoma. Clin Cancer Res.

[28] Ackermann S, Kocak H, Hero B, Ehemann V, Kahlert Y, Oberthuer A, Roels F, Theißen J, Odenthal M, Berthold F, Fischer M (2014). FOXP1 inhibits cell growth and attenuates tumorigenicity of neuroblastoma. BMC Cancer.

[29] Yáñez Y, Grau E, Rodríguez-Cortez VC, Hervás D, Vidal E, Noguera R, Hernández M, Segura V, Cañete A, Conesa A, Font de Mora J, Castel V (2015). Two independent epigenetic biomarkers predict survival in neuroblastoma. Clin Epigenetics.

[30] Ram Kumar RM, Boro A, Fuchs B (2016). Involvement and Clinical Aspects of MicroRNA in Osteosarcoma. Int J Mol Sci.

[31] Walz S, Lorenzin F, Morton J, Wiese KE, von Eyss B, Herold S, Rycak L, Dumay-Odelot H, Karim S, Bartkuhn M, Roels F, Wüstefeld T, Fischer M (2014). Activation and repression by oncogenic MYC shape tumour-specific gene expression profiles. Nature.

[32] Augoff K, McCue B, Plow EF, Sossey-Alaoui K (2012). miR-31 and its host gene lncRNA LOC554202 are regulated by promoter hypermethylation in triple-negative breast cancer. Mol Cancer.

[33] Parodi F, Carosio R, Ragusa M, Di Pietro C, Maugeri M, Barbagallo D, Sallustio F, Allemanni G, Pistillo MP, Casciano I, Forlani A, Schena FP, Purrello M (2016). Epigenetic dysregulation in neuroblastoma: A tale of miRNAs and DNA methylation. Biochim Biophys Acta.

[34] Maugeri M, Barbagallo D, Barbagallo C, Banelli B, Di Mauro S, Purrello F, Magro G, Ragusa M, Di Pietro C, Romani M, Purrello M (2016). Altered expression of miRNAs and methylation of their promoters are correlated in neuroblastoma. Oncotarget.

[35] Das S, Bryan K, Buckley PG, Piskareva O, Bray IM, Foley N, Ryan J, Lynch J, Creevey L, Fay J, Prenter S, Koster J, van Sluis P (2013). Modulation of neuroblastoma disease pathogenesis by an extensive network of epigenetically regulated microRNAs. Oncogene.

[36] Buechner J, Tømte E, Haug BH, Henriksen JR, Løkke C, Flægstad T, Einvik C (2011). Tumour-suppressor microRNAs let-7 and mir-101 target the proto-oncogene MYCN and inhibit cell proliferation in MYCN-amplified neuroblastoma. Br J Cancer.

[37] Lynch J, Fay J, Meehan M, Bryan K, Watters KM, Murphy DM, Stallings RL (2012). MiRNA-335 suppresses neuroblastoma cell invasiveness by direct targeting of multiple genes from the non-canonical TGF-β signalling pathway. Carcinogenesis.

[38] Romania P, Bertaina A, Bracaglia G, Locatelli F, Fruci D, Rota R (2012). Epigenetic deregulation of microRNAs in rhabdomyosarcoma and neuroblastoma and translational perspectives. Int J Mol Sci.

[39] Foley NH, Bray IM, Tivnan A, Bryan K, Murphy DM, Buckley PG, Ryan J, O’Meara A, O’Sullivan M, Stallings RL (2010). MicroRNA-184 inhibits neuroblastoma cell survival through targeting the serine/threonine kinase AKT2. Mol Cancer.

[40] Takwi AA, Wang YM, Wu J, Michaelis M, Cinatl J, Chen T (2014). miR-137 regulates the constitutive androstane receptor and modulates doxorubicin sensitivity in parental and doxorubicin-resistant neuroblastoma cells. Oncogene.

[41] Li KK, Li F, Li QS, Yang K, Jin B (2013). DNA methylation as a target of epigenetic therapeutics in cancer. Anticancer Agents Med Chem.

[42] George RE, Lahti JM, Adamson PC, Zhu K, Finkelstein D, Ingle AM, Reid JM, Krailo M, Neuberg D, Blaney SM, Diller L (2010). Phase I study of decitabine with doxorubicin and cyclophosphamide in children with neuroblastoma and other solid tumors: a Children’s Oncology Group study. Pediatr Blood Cancer.

[43] Westerlund I, Shi Y, Toskas K, Fell SM, Li S, Surova O, Södersten E, Kogner P, Nyman U, Schlisio S, Holmberg J (2017). Combined epigenetic and differentiation-based treatment inhibits neuroblastoma tumor growth and links HIF2α to tumor suppression. Proc Natl Acad Sci USA.

[44] Hattori N, Mori A, Kimura K, Kubo E, Asada K, Kawamoto H, Ushijima T

[45] Wong M, Tee AE, Milazzo G, Bell JL, Poulos RC, Atmadibrata B, Sun Y, Jing D, Ho N, Ling D, Liu PY, Zhang XD, Hüttelmaier S (2017). The Histone Methyltransferase DOT1L Promotes Neuroblastoma by Regulating Gene Transcription. Cancer Res.

[46] Veschi V, Liu Z, Voss TC, Ozbun L, Gryder B, Yan C, Hu Y, Ma A, Jin J, Mazur SJ, Lam N, Souza BK, Giannini G (2017). Epigenetic siRNA and Chemical Screens Identify SETD8 Inhibition as a Therapeutic Strategy for p53 Activation in High-Risk Neuroblastoma. Cancer Cell.

[47] Ke XX, Zhang D, Zhu S, Xia Q, Xiang Z, Cui H (2014). Inhibition of H3K9 methyltransferase G9a repressed cell proliferation and induced autophagy in neuroblastoma cells. PLoS One.

[48] Shi Y (2007). Histone lysine demethylases: emerging roles in development, physiology and disease. Nat Rev Genet.

[49] Yang J, Milasta S, Hu D, AlTahan AM, Interiano RB, Zhou J, Davidson J, Low J, Lin W, Bao J, Goh P, Nathwani AC, Wang R (2017). Targeting histone demethylases in MYC-driven neuroblastomas with ciclopirox. Cancer Res.

[50] Amente S, Milazzo G, Sorrentino MC, Ambrosio S, Di Palo G, Lania L, Perini G, Majello B (2015). Lysine-specific demethylase (LSD1/KDM1A) and MYCN cooperatively repress tumor suppressor genes in neuroblastoma. Oncotarget.

[51] Eckschlager T, Plch J, Stiborova M, Hrabeta J (2017). Histone Deacetylase Inhibitors as Anticancer Drugs. Int J Mol Sci.

[52] Rettig I, Koeneke E, Trippel F, Mueller WC, Burhenne J, Kopp-Schneider A, Fabian J, Schober A, Fernekorn U, von Deimling A, Deubzer HE, Milde T, Witt O, Oehme I (2015). Selective inhibition of HDAC8 decreases neuroblastoma growth *in vitro* and *in vivo* and enhances retinoic acid-mediated differentiation. Cell Death Dis.

[53] Bayat Mokhtari R, Baluch N, Ka Hon Tsui M, Kumar S, S Homayouni T, Aitken K, Das B, Baruchel S, Yeger H (2017). Acetazolamide potentiates the anti-tumor potential of HDACi, MS-275, in neuroblastoma. BMC Cancer.

[54] Almeida VR, Vieira IA, Buendia M, Brunetto AT, Gregianin LJ, Brunetto AL, Klamt F, de Farias CB, Abujamra AL, Lopez PL, Roesler R (2017). Combined Treatments with a Retinoid Receptor Agonist and Epigenetic Modulators in Human Neuroblastoma Cells. Mol Neurobiol.

[55] Cohen I, Poręba E, Kamieniarz K, Schneider R (2011). Histone modifiers in cancer: friends or foes?. Genes Cancer.

[56] Gajer JM, Furdas SD, Gründer A, Gothwal M, Heinicke U, Keller K, Colland F, Fulda S, Pahl HL, Fichtner I, Sippl W, Jung M (2015). Histone acetyltransferase inhibitors block neuroblastoma cell growth *in vivo*. Oncogenesis.

[57] Ruiz-Pérez MV, Henley AB, Arsenian-Henriksson M (2017). The MYCN protein in health and disease. Genes (Basel).

[58] Yang J, AlTahan AM, Hu D, Wang Y, Cheng PH, Morton CL, Qu C, Nathwani AC, Shohet JM, Fotsis T, Koster J, Versteeg R, Okada H (2015). The role of histone demethylase KDM4N in Myc signaling in neuroblastoma. J Natl Cancer Inst.

[59] Liu PY, Atmadibrata B, Mondal S, Tee AE, Liu T (2016). NCYM is upregulated by lncUSMycN and modulates N-Myc expression. Int J Oncol.

[60] Suenaga Y, Islam SM, Alagu J, Kaneko Y, Kato M, Tanaka Y, Kawana H, Hossain S, Matsumoto D, Yamamoto M, Shoji W, Itami M, Shibata T (2014). NCYM, a Cis-antisense gene of MYCN, encodes a de novo evolved protein that inhibits GSK3β resulting in the stabilization of MYCN in human neuroblastomas. PLoS Genet.

[61] Sun Y, Bell JL, Carter D, Gherardi S, Poulos RC, Milazzo G, Wong JW, Al-Awar R, Tee AE, Liu PY, Liu B, Atmadibrata B, Wong M (2015). WDR5 supports an N-Myc transcriptional complex that drives a protumorigenic gene expression signature in neuroblastoma. Cancer Res.

[62] Fabian J, Opitz D, Althoff K, Lodrini M, Hero B, Volland R, Beckers A, de Preter K, Decock A, Patil N, Abba M, Kopp-Schneider A, Astrahantseff K (2016). MYCN and HDAC5 transcriptionally repress CD9 to trigger invasion and metastasis in neuroblastoma. Oncotarget.

[63] Peifer M, Hertwig F, Roels F, Dreidax D, Gartlgruber M, Menon R, Krämer A, Roncaioli JL, Sand F, Heuckmann JM, Ikram F, Schmidt R, Ackermann S (2015). Telomerase activation by genomic rearrangements in high-risk neuroblastoma. Nature.

